# On-demand assembly of polymeric nanoparticles for longer-blood-circulation and disassembly in tumor for boosting sonodynamic therapy

**DOI:** 10.1016/j.bioactmat.2022.03.009

**Published:** 2022-03-12

**Authors:** Mei Wen, Nuo Yu, Shiwen Wu, Mengmeng Huang, Pu Qiu, Qian Ren, Meifang Zhu, Zhigang Chen

**Affiliations:** State Key Laboratory for Modification of Chemical Fibers and Polymer Materials, College of Materials Science and Engineering, Donghua University, Shanghai, 201620, China

**Keywords:** Polymeric nanoparticles, On-demand assembly, Longer-blood-circulation, On-demand disassembly, Sonodynamic therapy

## Abstract

Sonodynamic therapy (SDT) is one of the promising strategies for tumor therapy, but its application is usually hindered by fast clearance in blood-circulation, abnormal tumor microenvironment, and inefficient generation of reactive oxygen species. To solve these problems, we proposed an on-demand assembly-disassembly strategy, where the assembly is favorable for longer-blood-circulation and then the disassembly in tumor is favorable for boosting SDT. Hematoporphyrin monomethyl ether (HMME) as the model of organic sonosensitizers were conjugated with hyaluronic acid (HA). Then HA-HMME was mixed with catalase (CAT) and assembled into polymeric nanoparticles (CAT@HA-HMME NPs) with size of ∼80 nm. CAT@HA-HMME NPs exhibit good biocompatibility and a longer blood half-time (t_1/2_ = 4.17 h) which is obviously longer than that (∼0.82 h) of HMME molecules. After HA receptor-mediated endocytosis of cancer cells, CAT@HA-HMME NPs can be cleaved by endogenous hyaluronidase, resulting in the on-demand disassembly in tumor to release HA-HMME molecules and CAT. The CAT catalyzes the endogenous H_2_O_2_ into O_2_ to relieve the hypoxic microenvironment, and the released HA-HMME exhibits a higher ROS generation ability, greatly boosting SDT for the inhibition of tumor growth. Therefore, the on-demand assembly-disassembly strategy may provide some insight in the design and development of nanoagents for tumor therapy.

## Highlights

• On-demand assembly from molecules to nanoparticles for longer-blood-circulation.
• On-demand disassembly in presence of hyaluronidase (in tumor) for boosting sonodynamic effects.
• Efficient damage on cancer cells *in-vitro* and Significant inhibition of the tumor growth due to the enhanced SDT.

## Graphical abstract

**Figure:**
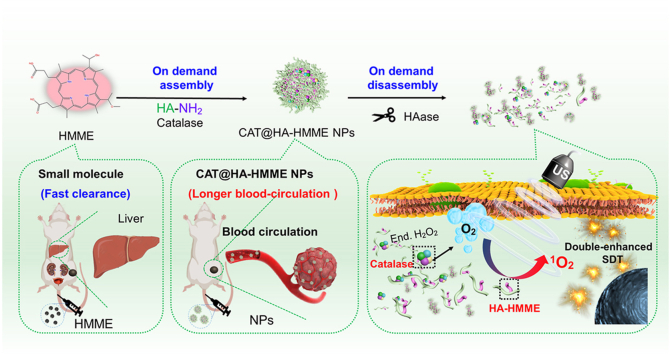

## 1 Introduction

Ultrasound (US)-excited sonodynamic therapy (SDT) is regarded as an alternative treatment technology to overcome the limited penetration depth of phototherapy (such as photo-thermal therapy (PTT) and photo-dynamic therapy (PDT)) [1,2]. The SDT relies on the reactive oxygen species (ROS) emanating from sonosensitizers upon US irradiation, and thus the key to SDT is to develop sonosensitizers. Two kinds of sonosensitizers have been developed. One is the inorganic sonosensitizers (such as MnWO_x_ [2], TiO_2_ [3] and noble metal [4] based nanoparticles), and they are widely studied on account of their stability. But their degradation *in vivo* is difficult, which hinders the practical application. The other is organic sonosensitizers, and they usually have relatively higher biocompatibility and more potential in the clinical application. Most organic sonosensitizers are derived from photosensitizers, which are porphyrin and its derivatives (such as protoporphyrin [5], IR780 [6], hematoporphyrin monomethyl ether (HMME) [7]). The porphyrins have a high quantum yield in the ROS generation. However, these traditional organic sonosensitizers usually suffer from some limitations, such as low water solubility, fast metabolism and fast clearance from blood circulation. These limitations induce inadequate pharmacokinetics and the insufficient SDT efficiency [8]. Therefore, it is of paramount importance to develop new strategies to realize efficient SDT with organic sonosensitizers.

Fortunately, the development of nanomedicines supplies a possible pathway to address the problem of limited pharmacokinetics. When organic molecules are assembled into nanoparticles (NPs), the assembled NPs usually show the good water solubility, slow metabolism and especially longer blood-circulation time than their parent small molecules [9]. For instance, DNA tetrahedral nanoparticles (∼7.5 nm) exhibited a longer blood half-life (t_1/2_ ≈24.2 min) than the siRNA (t_1/2_ ≈6 min) [9]. Plenty of researches have demonstrated that nanoparticles with suitable sizes (20–200 nm) can circulate for longer times in the bloodstream and have a greater chance to reach the tumor site [10,11]. Thus, the key of nanomedicines is to develop nanoparticles containing imaging and/or therapy molecules. Several works have focused on the conversion of sensitizers into organic nanostructures (such as polymeric nanoparticles [12]). For example, hyaluronic acid (HA) as the major component of extracellular matrix is implicated in cell-cell and cell-matrix interactions. It is widely known to specifically bind with cluster determinant 44 (CD44) that is over-expressed in many different types of cancer cells [13], including ovarian cancer cells (SK-OV3) and colon cancer cells (CT26). Meanwhile, HA has multiple functional groups (such as carboxyl, and hydroxyl groups) which are available for chemical conjugation of other drugs molecules [14]. HA-conjugated drugs molecules can easily self-assemble into nanoparticles [15], and the nanoparticles can maintain the good stability and prevent the leakage of the drugs during the systemic circulation. Some photosensitizers have also been well encapsulated into the hydrophilic HA-based nanoparticles, and the hydrophilic shells of nanoparticles allow the extension of the plasma half-life and enhance cellular internalization [16,17]. Therefore, these assembled NPs have become a hot topic of research.

Unfortunately, the self-assembled NPs may also pose a double-edged sword, since the high local concentrations of organic sensitizers in NPs can cause severe aggregation-induced quenching (ACQ) and subsequently reduced therapeutic effect. For example, zinc(II) phthalocyanine molecules exhibit a strong fluorescence emission at 691 nm, while the resulting NPs have dramatically diminished the fluorescence intensity and thus weaker PDT effects due to ACQ [18]. For SDT, the assembled Fe-HMME NPs also exhibit weaker fluorescence as well as decreased ^1^O_2_ generation ability under US irradiation, compared to free HMME sonosensitizers [19]. To improve the therapeutic effect, there are two feasible methods. One is to improve the sensitization ability of sonosensitizer itself by decomposing nanoparticles. For example, HA-based nanoparticles can be rapidly degraded by the hyaluronidases (HAase) which is abundant in the cytosol of tumor cells and may enable the release of inner cargos [20]. Yang group [21] has taken advantage of HAase to trigger the disassembly to release the quenched photosensitizer molecules, resulting in fluorescence activation. The other is to supply abundant oxygen since hypoxia tumor microenvironment (TME) has an adverse effect on O_2_-dependent PDT or SDT [19]. Interestingly, it is feasible that endogenous H_2_O_2_ can generate oxygen within the tumor with catalysts. Among the catalysts, catalase (CAT) from the bovine liver can effectively convert H_2_O_2_ to O_2_ in the tumor site. Lei group [22] reported a CAT-encapsulating MIL-101 to deal with a challenge of hypoxia issues in PDT. Therefore, both disassembly and CAT encapsulation strategies have been developed to improve the PDT effects. However, there are few reports for simultaneously improving SDT effects and pharmacokinetics.

**Fig. 1:**
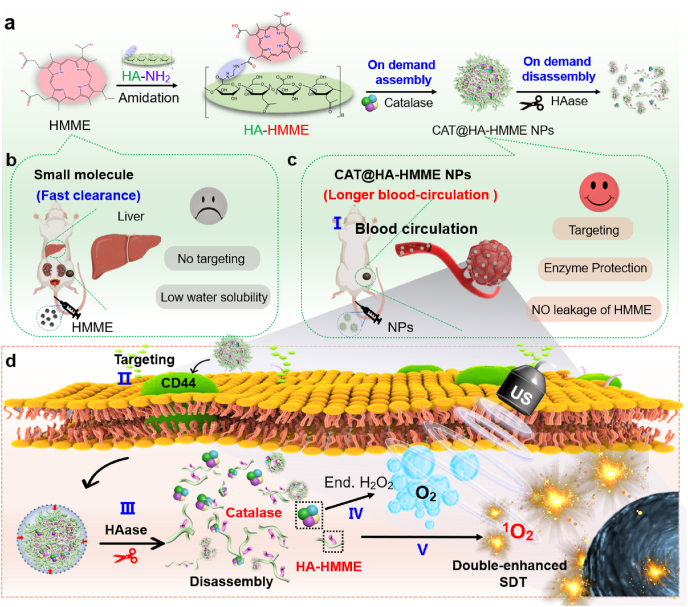
On-demand assembly-disassembly strategy. (a) Schematic illustration of the assembly and disassembly process of CAT@HA-HMME NPs. (b) Small molecules with fast clearance feature. (c) The assembled CAT@HA-HMME NPs with longer blood-circulation. (d) On-demand disassembly of CAT@HA-HMME NPs in cancer cells for boosting SDT.

It can be expected that the ideal sonosensitizers should be nanoparticles with the suitable sizes (20–100 nm), where they exhibit a long blood half-life after injection into the blood, and when they reach the tumor region, they become small free molecules to release drugs, sensitizers and/or catalysts for boosting SDT effect. To obtain such ideal sonosensitizers, we proposed an on-demand assembly-disassembly strategy, where the assembly is favorable for longer-blood-circulation and then the disassembly in tumor is favorable for enhanced SDT. As the model of organic sonosensitizers, HMME usually suffers from low water solubility and fast metabolism [23]. When HMME solution is intravenously injected, HMME can be rapidly eliminated from blood circulation (blood half-time: ∼0.82 h) and then executed in liver [24] (Fig. 1a and b). Meanwhile, pure HMME cannot specifically accumulate in the tumor site due to the lack of targeting ability. To solve these issues, HMME conjugated hyaluronic acid (HA) and catalase (CAT) were assembled into into polymeric NPs (CAT@HA-HMME NPs) (Fig. 1a, c). After the assembly, the CAT@HA-HMME NPs with the average diameter of ∼102.5 nm confer a longer blood half-time (t_1/2_ = 4.17 h), good targeting and enzyme-protection abilities. After HA receptor-mediated endocytosis of cancer cells, CAT@HA-HMME NPs can be cleaved by the HAase, resulting in the disassembly process in tumor to release HA-HMME molecules and CAT (Fig. 1d). The disassembly of CAT@HA-HMME NPs can efficiently alleviate the aggregation-casued reduction of ^1^O_2_, improving the sensitization ability of sonosensitizer itself. The CAT depletes endogenous H_2_O_2_ into O_2_ to relieve the hypoxic TME and to boost the production of cytotoxic ^1^O_2_ for SDT. The improvement of sonosensitizer and increased O_2_ concentration doubly enhance ^1^O_2_ generation and induce irreversible oxidation of cancer cells.

## 2 Materials and methods

### 2.1 Chemicals

Hemoporfin (HMME) was received from Shanghai Xianhui Pharmaceutical Co., Ltd. Hydrogen peroxide (H_2_O_2_), N·N-dimethylformamide (DMF), and 1.3-diphenyl benzofuran (DPBF), 1-ethyl-3(3-dimethylaminopropyl) carbodiimide (EDC), N-hydroxysuccinimide (NHS) were brought from Sinopharm Chemical Reagent Co., Ltd. HAase (300 U/mg) was obtained from Sigma-Aldrich LLC. Catalase (CAT) was acquired from Shanghai Yanyi Biotechnology Corporation. Hyaluronic acid (HA, MW: 200 k) was brought from Jiangsu haihua biotechnology co. LTD. Tris(4,7-diphenyl-1,10-phenanthroline) ruthenium (II) dichloride complex ([Ru(dpp)_3_]^2+^Cl_2_) was from Energy Chemical. Fetal bovine serum (FBS), dulbecco's modified eagle medium (DMEM), 0.05% trypsin-EDTA, Phosphate buffered saline (PBS, pH 7.4) and penicillin-streptomycin solution were obtained from Gibco (NY, USA).

### 2.2 Preparation of CAT@HA-HMME NPs

**Synthesis of HA-NH**_**2**_**:** HA (850 mg, 4.0 μmol, 2108 μmol of carboxyl group) was dissolved in 10 mL of PBS, then its carboxyl group activated by EDC (2108 μmol) and NHS (2108 μmol). After the reaction lasted for 2 hours, the above solution was added by 1 mL DMSO containing ethylenediamine (18 mg), followed by stirred for overnight in darkness. Purification was further executed by dialysis against water for 2 days (MWCO 10 kDa), followed by freeze-drying to obtain HA-NH_2_. The extent of amine substitution on HA was determined by ^1^H NMR (400 MHz, D_2_O). The characteristic peaks of HA and ethylenediamine appear at 1.90 ppm (methyl proton of –NCOCH_3_ group) and 2.70–3.00 ppm (methylene groups).

**Synthesis of HA-HMME:** HMME was chemically conjugated onto HA as follows. 5 mg of HMME in 5 ml DMSO was activated by NHS (3.6 mg) and EDC (6.1 mg) for 2 h. Then, 5 mL PBS containing HA-NH_2_ (20 mg) was added to the above DMSO solution. The resulting solution was stirred overnight at room temperature. Next, the purification was carried out by filtration through dialysis (MWCO, 3500 Da) in ethanol/water (1:1, v/v) for 24 h.

**Assembly of CAT@HA-HMME NPs:** HA-HMME (5 mg) was added into PBS (5 mL) containing CAT (0.5 mL, ≥30,000 units/mL). Then the solution was ultrasonicated with a probe-type sonicator (Ultrasonic Homogenizer) for 15 min in an ice bath. After, the dispersion was stirred for 12 h at 4 °C. In addition, to prepare fluorescein-labeled CAT@HA-HMME NPs (F-CAT@HA-HMME NPs), CAT was firstly labeled with FITC. FITC-labeled CAT was synthesized by FITC (100 μL, 0.1 mg/mL) reacting with CAT (0.5 mL, ≥30,000 units/ml) in 5 ml sodium carbonate buffer (pH = 9.0) for 12 h, followed dialyzed with PBS for 2 days.

### 2.3 Characterization

**Physical characterization.** CAT@HA-HMME NPs were analyzed by using transmission electron microscopy (TEM, FEI Talos F200S), Fluorescence Spectrophotometer (JASCO, FP-6600), and UV-vis-NIR absorption spectrophotometer (Shimadzu UV-3600).

^**1**^**O**_**2**_**production.** The ^1^O_2_ generation potential of CAT@HA-HMME NPs was determined using DPBF. 20 μL of DPBF (2 mg/mL) was added into 3 mL CAT@HA-HMME NPs (10 μg/mL), which was in water-bath at 37 °C. After different US irradiation (40 kHz, 2.5 W/cm^2^) durations in the dark, the absorbance changes of DPBF at 416 nm were monitored to quantify the ^1^O_2_ generation rate from CAT@HA-HMME NPs by UV–vis spectroscopy. For comparison, the effect of H_2_O_2_ (1 mM) or HAase (20 μg/mL) on ^1^O_2_ generation from CAT@HA-HMME NPs was also assessed upon US irradiation. And the rate constant for ^1^O_2_ generation was calculated using the following equation: ln([DPBF]_t_/[DPBF]_0_) = -kt.

**Evaluation of catalase activity.** The catalytic activities of CAT@HA-HMME NPs were evaluated by the standard Gόoth's method [25]. Briefly, free CAT or CAT@HA-HMME NPs was mixed with 0.5 mL solution of H_2_O_2_ (50 mM), respectively, and reacted for 60 s at 37 °C. Next, the reaction was terminated by the addition of ammonium molybdate (0.5 mL, 32.4 mM), which could react with the residual H_2_O_2_ to form stable yellow complexes. After the centrifugation, the absorbance at 400 nm was recorded by UV–vis spectrometer to measure the catalase activity. For assessing the stability of catalase against protease digestion, free catalase and CAT@HA-HMME NPs at the same CAT concentration (0.09 mg/mL) were incubated with protease K (0.4 mg/mL) at 37 °C. At predetermined time points, samples were removed for catalase activity assay. In addition, the dissolved O_2_ was measured in the 1 mM of H_2_O_2_ solution by the portable dissolved oxygen meter.

### 2.4 Cellular experiments

**Cell culture.** SK-OV3, CT26 and Human Umbilical Vein Endothelial Cells (HUVEC) were purchased from Type Culture Collection of the Chinese Academy of Sciences, Shanghai, China. All cells were cultured in DMEM medium containing 10% FBS and 1% penicillin-streptomycin in the presence of 5% CO_2_ at 37 °C.

**Cellular uptake.** CT26 or SK-OV3 cells were seeded into a confocal dish and incubated with CAT@HA-HMME NPs (100 μg/mL) for 6 h, and then the cells were washed with PBS to removed residual CAT@HA-HMME NPs. For comparison, the HA pretreated cells were also incubated with CAT@HA-HMME NPs at the same condition. After that, the cells were stained with 4,6-diamino-2-phenylindole (DAPI) for 20 min before confocal imaging.

**Intracellular O**_**2**_**detection.** The intracellular O_2_ level was detected by an optical probe and immunostaining. For the optical probe, the CT26 cells were seeded into dishes and cultured in a hypoxia atmosphere (1% O_2_) for 24 h. Then, the cells were incubated with [Ru(dpp)_3_]^2+^Cl_2_ (10 μg/mL) for 6 h, followed by washing with PBS. The CT26 cells were then co-incubated with CAT@HA-HMME NPs (100 μg/mL) for 12 h. The intracellular oxygen level was assayed by detecting the luminescence intensity of Ru(dpp)_3_]^2+^Cl_2_. To further evaluate hypoxic level, the hypoxia-induced factor 1α (HIF-1α) expression was investigated by immunostaining. The CT26 cells in a hypoxia atmosphere (1% O_2_) were incubated with HA-HMME NPs (100 μg/mL) and CAT@HA-HMME NPs (100 μg/mL) for 12 h and fixed by 4% paraformaldehyde for 20 min, followed by washing with PBS. Then the cells were treated with 0.1% Triton X-100 and washed by 0.1% Tween-20. After blocking buffer was introduced for 1 h, the primary anti-HIF-1α antibody at the concentration of 2 μg/mL was incubated with the cells at 37 °C for 1.5 h. Subsquently, the cells were treated with secondary Alexa Fluor488-conjugated goat anti-rabbit IgG H&L antibody at the 5 μg/mL concentration in darkness for 1 h. For comparios, the cells in the a hypoxia atmosphere and in a normoxia atmosphere (21% O_2_) were also stained with immunostaining. Before imaging, the cells were stained with DAPI (300 μM) for 20 min.

**Assessment of cytotoxicity.** CT26, SK-OV3, and HUVEC cells were seeded into 96-wells at a density of ∼1 × 10^4^. After incubation for 12 h, the medium was replaced by fresh media containing CAT@HA-HMME NPs at a series of final concentrations (0–200 μg/mL). After 24 h incubation, the medium was removed, and the cells were washed with PBS three times. For assessing the cell proliferation, Cell Counting Kit-8 (CCK-8) assay was employed to culture with cells for 4 h. Then the absorbance at 450 nm was measured using a microplate reader.

**Intracellular**^**1**^**O**_**2**_**detection.** The SDT efficacy of CAT@HA-HMME NPs was measured by detecting the intracellular generation of ROS. In brief, SK-OV3 cells as a model were seeded into dishes at a density of 1 × 10^5^ cells. After incubation for 12 h, the SK-OV3 were treated with CAT@HA-HMME NPs at the concentration of 100 μg/mL. Next, the cells were dyed by DCFH-DA for 1 h and treated by US irradiation (40 kHz, 2.5 W/cm^2^) for 3 min, followed by washing with PBS twice. Finally, the cells were observed by a fluorescence microscope.

### 2.5 Animal experiment

**Tumor model.** BALB/c mice (6–8 weeks of age) were obtained from Shanghai SLAC Laboratory Animal Center. All animal investigations were conformed to the guide for the Care and Use of Laboratory Animals by the U.S. National Institutes of Health (NIH Publication no. 86–23, revised 1985) and performed in accordance with the protocols approved by the Animal Welfare and Research Ethics Committee of Donghua University. CT26 cells (3 × 10^6^/mouse) were injected into the back of the mouse to build the tumor model. All experiments did not start until the tumors were grown to a volume of 100–150 mm^3^.

**Hemolysis assay.** Blood (10 mg) was obtained from BALB/c mice. The red blood cells (RBCs) were separated by centrifugation at 2000 rpm for 15 min and washed by PBS. Then, 6 mL of PBS was added into RBCs and the diluted RBCs were divided into six equal parts. Subsequently, each RBCs sample was centrifugated and added to 1 mL of water (positive control), PBS (negative control), and different CAT@HA-HMME NPs of different concentrations, respectively. After incubation for 1 h, all the samples were centrifugated at 12,000 rpm for 15 min, and the absorbance of the supernatant at 576 nm was measured using UV–vis spectrometer. And the percentage of hemolysis was calculated by the following formula: Hemolysis ratio (%) = (A _sample_ - A _negative_)/(A _positive_ - A _negative_) × 100%

**Pharmacokinetic analysis.** For blood circulation, BALB/c mice were intravenously injected with pure HMME or CAT@HA-HMME NPs (5 mg/kg). The blood samples (10 mg) were collected at different time points after injection. 100 μL of nitric acid was added to each sample and the samples were centrifuged at 12,000 rpm for 15 min. The quantity of HMME in blood samples was determined by FL spectra (subtracting auto-fluorescence from the blood sample of an untreated mouse) [26]. The concentrations of HMME were calculated as the percentage of injected dose gram of tissue (%ID/g). The circulation half-lives (t_1/2_) were calculated by third-order exponential fitting.

**In vivo imaging.** For *in vivo* fluorescence imaging, the CAT@HA-HMME NPs were pre-labeled with Cy5.5 according to the previous report [27]. Then the labeled CAT@HA-HMME NPs (in PBS) was injected into the tail vein of mice, and the images were acquired from IVIS Lumina II at various time points (0, 1, 2, 4, 8, 10 and 12 h).

***In vivo* ROS generation.** The detection of ROS generation was executed according to the previous reports [28]. Briefly, the HA-HMME NPs or CAT@HA-HMME NPs were intravenously injected into CT26 tumor-bearing mice. After 12 h, the 50 μL of DCFH-DA solution (20 μM) was intratumorally injected. After 30 min, the tumors were irradiated by US ((1.0 MHz, 2.5 W/cm^2^, 10 min, 25% duty cycle). Then, the treated tumors were harvested and fixed by 4% paraformaldehyde solution, followed by section. Before imaging, the sections were stained by DAPI.

**SDT therapy *in vivo*.** Mice bearing CT26 tumors were divided into four groups: (Ⅰ) PBS; (II) CAT@HA-HMME NPs; (III) HA-HMME NPs + US; (IV) CAT@HA-HMME NPs + US. Group II-IV were administered by intravenous injection at an equal HMME dose of 9.8 mg/kg on day 1 and day 5. After 24 h of injection, mice received US treatment (1.0 MHz, 2.5 W/cm^2^, 10 min, 25% duty cycle). Tumor size and weight were measured every two days. The tumor volumes were calculated by using the formula: volume = length × width^2^/2. For hematoxylin and eosin (H&E) staining, the tumor tissues and the major organs were extracted, followed by fixing in 4% paraformaldehyde. And the slices were stained by H&E.

## 3 Results and discussion

### 3.1 Assembly and characterization of CAT@HA-HMME NPs

**Fig. 2:**
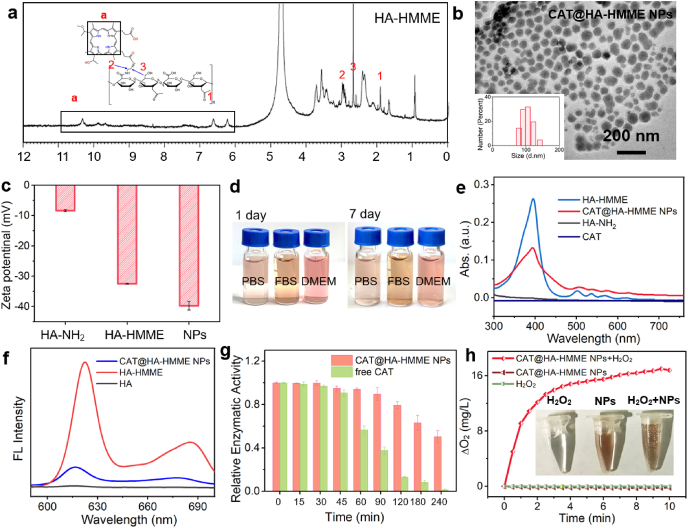
On-demand assembly of CAT@HA-HMME NPs. (a) ^1^H NMR of HA-HMME. (b) TEM image and DLS of CAT@HA-HMME NPs. (c) Zeta potential of HA-NH_2_, HA-HMME, and CAT@HA-HMME NPs. (d) Photographs of CAT@HA-HMME NPs in different biological fluids. (e) UV–vis spectra of CAT, HA-NH_2_, HA-HMME, and CAT@HA-HMME NPs. (f) Fluorescence spectra of HA-NH_2_, HA-HMME, and CAT@HA-HMME NPs. (g) Relative enzymatic activity of free catalase and CAT@HA-HMME NPs in the presence of proteinase K (h). Change in O_2_ concentration versus time for different samples. Inset: Macroscopic images of different samples.

CAT@HA-HMME NPs were prepared by the conjugation and encapsulation/assembly two-step strategy, as illustrated in Fig. 1a. In the first step, HMME was activated by EDC and NHS, and then conjugated onto the HA-NH_2_ via formation of a stable amide bond. The formation of the amphiphilic HA-HMME was investigated by ^1^H NMR spectra. The up field area of the ^1^H NMR spectra (*δ* = 1.90 ppm, *δ* = 3.20–4.30 ppm) can be assigned to the typical resonances of the hydrogen atoms of the HA (*δ* = 1.90 ppm, *δ* = 3.20–4.30 ppm, in the supporting information Fig. S1) [29], whereas that of ethylenediamine is found at the range of 2.70–3.00 ppm [29]. The other peaks in the downfield area (*δ* = 6.0–11.5 ppm) are attributed to the HMME section (Fig. 2a) [30]. ^1^H NMR spectrum confirms the conjugation of the HMME with HA. The second step was to add the HA-HMME into CAT solution, and then CAT could be encapsulated inside the hydrophobic core of amphiphilic HA-HMME during the ultrasonic process, resulting in the self-assembly formation of polymeric nanoparticles (namely, CAT@HA-HMME NPs). The formation of CAT@HA-HMME NPs was analyzed by transmission electron microscopy (TEM), dynamic light scattering (DLS) and Zeta potential. The TEM image reveals that the CAT@HA-HMME sample is composed of uniform nanospheres with size of ∼80 nm (Fig. 2b). DLS result (inset of Fig. 2b) shows that CAT@HA-HMME NPs have a narrow size distribution with hydrodynamic diameters of ∼102.5 nm, which is larger than the size (∼80 nm) from TEM due to the swelling of particles in water [7]. Before HMME conjugation, the HA-NH_2_ exhibits a Zeta potential of −8.4 mV (Fig. 2c). After HMME conjugation and CAT encapsulation, the resulting HA-HMME and CAT@HA-HMME NPs show a more negative zeta potential of −32.5 and −39.8 mV, respectively, which are consistent with the successive modification of HA-NH_2_ with the negative charge HMME [31] and negative CAT [32]. It is also considered that high surface potential (>30 mV or < −30 mV) is beneficial to the suspension of nanoparticles [14,33]. As a result, when CAT@HA-HMME NPs are dispersed in different biological fluids (including PBS, FBS, and DMEM), no obvious precipitations can be found after the storage for 7 days (Fig. 2d), demonstrating their good stability. These facts confirm the well formation of CAT@HA-HMME NPs with high dispersibility.

To further analyze optical properties, UV–vis and fluorescence spectra were used to investigate different solutions of CAT, HA-NH_2_, HMME-HA or CAT@HA-HMME NPs. The HA-NH_2_ and CAT solutions have no obvious photoabsorption in a wide range (300–700 nm, Fig. 2e) and the HA-NH_2_ solution exhibts no obvious fluorescence (Fig. 2f). The UV–vis spectrum of HA-HMME exhibits several characteristic peaks (Figs. 2e and S2a), including a strong Soret band at 390 nm and four weaker Q band peaks at 470–640 nm from HMME [7,19]. After assembly into CAT@HA-HMME NPs, UV–vis spectrum has a weaker but broadened characteristic peak at ∼390 nm, due to π-π stacking between HMME-HA molecules [34]. The conjugation efficiency of HMME is calculated by using the UV calibration curve at 623 nm (Figs. S2b and c), and it is ∼18.8% [35]. Furthermore, the HA-HMME solution exhibits a strong fluorescent peak at 622 nm and a relatively weak peak at 686 nm. After the assembly, HMME fluorescence (Fig. 2f) from CAT@HA-HMME NPs shows an obvious decrease, which should be attributed to the ACQ effect via π−π stacking [21]. In addition, to verify the presence of CAT, CAT can be pre-labeled with fluorescein isothiocyanate (FITC, green fluorescence) and then be assembled with the HA-HMME to form FITC-labeled-CAT@HA-HMME NPs. Under 488 nm light excitation, the fluorescent spectrum of FITC-labeled-CAT@HA-HMME NPs (Fig. S2d) is featured with three emission peaks at 518, 613, and 674 nm, which are respectively indexed to FITC (518 nm) and HMME (613 and 674 nm) [36] characteristic peaks. Meanwhile, the encapsulation capability of CAT was determined by bicinchoninic acid (BCA) protein assay, and it is calculated to be 22.5% (Fig. S3a). These facts confirm that the CAT@HA-HMME NPs have the relatively decreased photoabsorption and fluorescence due to the assembly, and CAT can be well encapsulated.

It is well known that during SDT process, the therapeutic effect of sonosensitizers is highly dependent on the ability of ^1^O_2_ production and the surrounding environment, and it is always impaired by oxygen consumption and poorly organized vascular architecture. Aberrant metabolism of cancer cells causes the production of endogenous H_2_O_2_. The decomposition of H_2_O_2_ can be catalyzed by CAT to produce O_2_, which can realize an abundant O_2_ environment for O_2_-dependent SDT [19]. Herein, the catalytic ability of CAT@HA-HMME NPs was evaluated by the Gόoth method [25]. As shown in Fig. S3b, compared to free CAT (1.23), the CAT in the CAT@HA-HMME NPs has relatively low initial catalase activity (1.41), which is attributed to the reduction of reaction surface area after the encapsulation. It should be noted that the CAT is encapsulated into the CAT@HA-HMME NPs in the PBS through physical forces rather than chemical bonds. It would not destroy the structure and properties of the CAT without obvious effect on initial activity of CAT. Considering the existence of proteases in physiological environments [37], we assessed the enzymatic stability of CAT@HA-HMME NPs and free CAT against proteases (Fig. 2g). Free CAT is gradually digested and completely loses activity after incubation with protease K for 240 min. Unlike free CAT, the CAT encapsulated within CAT@HA-HMME NPs can be well protected and maintains ∼50.33% of its initial activity. The ability of O_2_ production is evaluated by a dissolved O_2_ meter (Fig. 2h). In the control groups (only CAT@HA-HMME NPs or only H_2_O_2_), O_2_ concentration is very low and no obvious change can be found during 10 min. After adding H_2_O_2_ (1 mM) into the CAT@HA-HMME NPs solution (0.8 mg/mL), the O_2_ concentration goes up rapidly, and a huge elevation (16.78 mg/L) can be found at 10 min. Additionally, the O_2_-enriched environment provided by CAT@HA-HMME NPs can also be demonstrated by the obvious bubble production (inset of Fig. 2h). Therefore, CAT@HA-HMME NPs can efficiently convert H_2_O_2_ to O_2_, with high enzymatic stability due to the protection of HA shell.

### 3.2 Disassembly-induced improvement of SDT

**Fig. 3:**
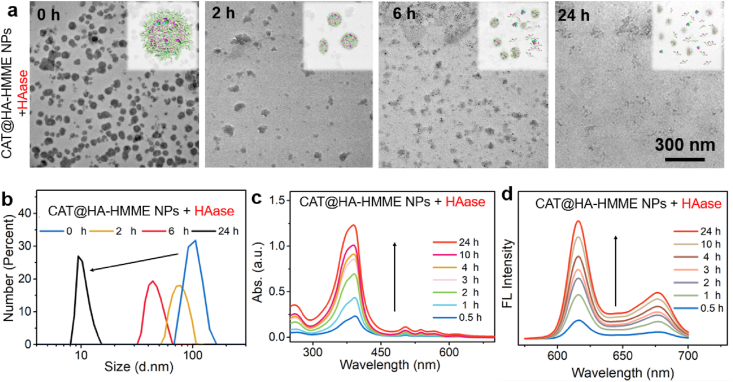
On-demand disassembly of CAT@HA-HMME NPs by HAase. Evolution of TEM images (a) and the size distributions (b) of CAT@HA-HMME NPs during the disassembly process by reacting with HAase for 0–24 h. Disassembly-induced elevation of photoabsorption (c) and fluorescence spectra (d) of the supernatant after centrifugation.

HAase as a spreading factor is overexpressed in many tumors, including breast and colon tumors. For example, the content of HAase (median activities) is 24.06–99.63 mU/g in metastatic breast cancer [38], while HAase is present at exceedingly low concentrations in human serum [39]. Meanwhile, the endogenous HAase degrades HA into oligosaccharide fragments at the tumor environment [21,39]. Thus, it can be expected that HA macromolecules in CAT@HA-HMME NPs can also be cleavaged by the endogenous HAase, probably resulting in the disassembly and then the release of HA-HMME. To verify this hypothesis, the disassembly process of CAT@HA-HMME NPs was investigated by adding HAase into CAT@HA-HMME NPs dispersion. To shorten reaction time, the concentration of HAase was determined to be 20 μg/mL (6 U/mL) according to the previous report [17]. After addition of HAase, the morphologies were analyzed by TEM. With the increase in time from 0 to 24 h, the original nanospheres are continuously corroded and disintegrated into fragments (Fig. 3a), and the size decreases from ∼102 nm to ∼28 nm (Fig. 3b). In addition, CAT@HA-HMME NP solution exhibits a photoabsorbance at ∼390 nm which is the characteristic peak of HMME (Fig. 2e). Interestingly, the absorbance at ∼390 nm from the supernatant of CAT@HA-HMME + HAase system goes up dramatically in 0–4 h and then increase slowly in the following 4–24 h (Fig. 3c, Fig. S3c). Meanwhile, fluorescent spectra reveal that the supernatant exhibits an increased intensity with the increased time from 0.5 to 24 h (Fig. 3d, Fig. S3d). Based on the above results, it can be concluded that HAase can decompose CAT@HA-HMME NPs, resulting in the efficient disassembly process and then the release of HA-HMME with stronger photoabsorption and fluorescence.

**Fig. 4:**
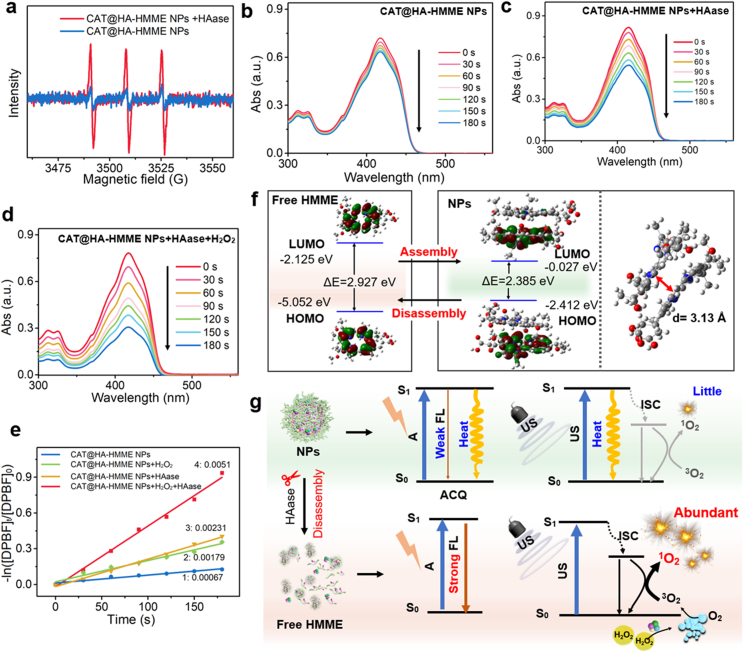
^1^O_2_ generation ability before and after the HAase-induced disassembly. (a) ESR spectra for US-triggered CAT@HA-HMME NPs and CAT@HA-HMME NPs + HAase. Time-dependent oxidation of DPBF indicating US-excited ^1^O_2_ generation in CAT@HA-HMME NPs solution (b), CAT@HA-HMME NPs + HAase solution (c), or CAT@HA-HMME NPs + HAase + H_2_O_2_ solution (d, after deducting the absorbance of CAT@HA-HMME NPs). (e) The ^1^O_2_ generation rate after different treatments. (f) The HOMO-LUMO orbital distribution of free HMME and NPs. (g) Schematic illustration of the proposed photoconversion routes and US-excitation production of ^1^O_2_ for CAT@HA-HMME NPs and their HAase-induced disassembled sample.

During HAase-induced disassembly process, the release of HA-HMME may also relieve the reduction of ROS causing by the aggregation, due to its higher photoabsorption and fluorescence. To investigate the generation ability of ^1^O_2_ under US irradiation, electron spin resonance (ESR) was firstly applied to monitor CAT@HA-HMME solution with or without HAase by using 2,2,6,6-Tetramethylpiperidine (TEMP) as a trapping agent. During the US irradiation process, CAT@HA-HMME NPs retain good stability, without obvious change in photoabsorption (Fig. S4a). After US irradiation for 5 min, the ESR spectrum of CAT@HA-HMME NPs (Fig. 4a) shows very weak signals, indicating the inefficient production of ^1^O_2_ due to ACQ effect. On the contrary, CAT@HA-HMME NPs + HAase system exhibits 1:1:1 triple signal, confirming the efficient production of ^1^O_2_, due to the disassembly of CAT@HA-HMME NPs by HAase. In addition, 1,3-diphenylisobenzofuran (DPBF) is also employed as a typical probe to detect ^1^O_2_ generation by UV–vis spectra. Under the US exposure, the CAT@HA-HMME NPs can induce the gradual decrease of absorbance of DPBF from 0.72 to 0.63 (Fig. 4b) with the low calculated rate constants of 0.00067 s^−1^ (Fig. 4e), suggesting the weak ability of ^1^O_2_ generation by CAT@HA-HMME NPs. After adding HAase (20 μg/mL) into CAT@HA-HMME NPs + DPBF solution, the calculated rate constant (0.00231 s^−1^) is 3.4 times that (0.00067 s^−1^) of only CAT@HA-HMME NPs group (Fig. 4c, e), indicating that HAase-induced disassembly can generate a higher amount of ^1^O_2_. In addition, if H_2_O_2_ (1 mM) is added into CAT@HA-HMME NPs + DPBF solution, the calculated rate constant (0.00179 s^−1^) is also higher than that of only CAT@HA-HMME NPs group (Fig. S4b), suggesting that CAT catalyzes the depletion of H_2_O_2_ into O_2_ to enhance SDT. Importantly, after simultaneous addition of H_2_O_2_ and HAase, the characteristic peaks of DPBF dramatically decrease from 0.78 to 0.31 (Fig. 4d), with the highest rate (0.0051 s^−1^, Fig. 4e) which is 7.6 times of that (0.00067 s^−1^) of only CAT@HA-HMME NPs group. Based on the above results, CAT@HA-HMME NPs can respond to US, but can only generate a little ^1^O_2_. While CAT@HA-HMME NPs can be disassembled by HAase to release CAT and HA-HMME. On the one hand, the CAT catalyzes H_2_O_2_ into O_2_, relieving hypoxia. On the other hand, HA-HMME can generate more ^1^O_2_ due to the disappearance of ACQ effect. As a result, the synergetic effects from both CAT and HA-HMME can greatly improve SDT effects.

To further analyze the difference in ^1^O_2_ yield before and after disassembly, we also carried out density functional theory (DFT) calculations with Gauss 16 program. The electron density of free HMME in the highest occupied molecular orbital (HOMO) and the lowest unoccupied molecular orbital (LUMO) concentrated on the porphyrin ring, with an energy gap of 2.927 eV (Fig. 4f). For CAT@HA-HMME NPs simulation, the energy gap of HOMO-LUMO is 2.385 eV, which is narrower than that of free HMME. HMME in CAT@HA-HMME NPs adopt a face-to-face stack mode, the distance between HMME molecules is 3.13 Å, indicating the existence of π–π interactions in the CAT@HA-HMME NPs. The strong π−π stacking leads to the significant ACQ effect [40]. As a result, CAT@HA-HMME NPs exhibit weaker photoabsorption (Fig. 2e) and fluorescence (Fig. 2f), as well as the aggregation-enhanced non-radiative transition (heat). Similarly, upon US irradiation, the strong nonradiative energy transfer [41] induces the reduction in ^1^O_2_ yield (Fig. 4g). Importantly, after the disassembly by HAase, the π−π stacking between HMME molecules is disrupted. The free HMME molecules have strong photoabsorption (Fig. 3c) and radiative transition (Fig. 3d). As a result, HMME molecules can also undergo an efficient ISC and result in a higher ^1^O_2_ yield during US irradiation process. Therefore, the on-demand disassembly of NPs is favorable for ^1^O_2_ generation and improves SDT.

### 3.3 Uptake of CAT@HA-HMME NPs and SDT *in vitro*

**Fig. 5:**
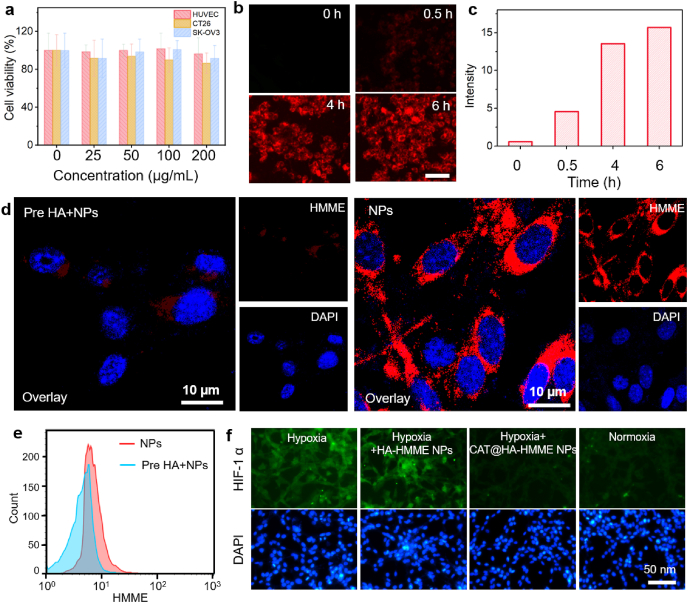
Uptake and oxygen level. (a) Cell viability of different cancer cell lines with CAT@HA-HMME NPs at 0–200 μg/mL for 24 h. (b) Time-dependent uptake. Scar bar is 50 μm. (c) Semi-quantitative analysis of fluorescence signals of HMME based on the images shown in Fig. 5b. CT26 cells uptake was assessed by CLSM (d) and flow cytometry (e). (f) Fluorescence images of CT26 cells immunostained to investigate hypoxic marker HIF-1α level after incubation with/without HA-HMME NPs andCAT@HA-HMME NPs.

The cytotoxicity of CAT@HA-HMME NPs were evaluated on different cell lines including SK-OV3, CT26, and HUVEC by Cell Counting Kit-8 (CCK-8) assay (Fig. 5a). After 24 h incubation, all groups demonstrate high cell viability (i.e. more than 86.5%) even at the CAT@HA-HMME NPs concentration up to 200 μg/mL. Meanwhile, SK-OV3 cells also co-culture with free HA (0–200 μg/mL) and HMME (0–50 μg/m) for 24 h. The cells remain high viability, indicating thier high biosafety (Figs. S5a and b). Therefore, the negligible cell cytotoxicity of CAT@HA-HMME NPs should be attributed to the clinically used HA and HMME, and biological catalase.

In addition, HA has specific affinity to CD44 that highly express in various cancer stem cells such as SK-OV3 and CT26 [42]. For example, SKOV3 are shown to have high CD44 expression (98.8%).As a result, CAT@HA-HMME may efficiently recognize and enter CD44-positive cells. Furthermore, HAase is abundant in the cytosol of tumor cells and it can efficiently cleave the HA in CAT@HA-HMME (Fig. 3a), conferring the disassembly of CAT@HA-HMME and then the release of HA-HMME and CAT for improving SDT (Fig. 4). To verify these facts *in vitro*, the CT26 and SKOV3 cells were incubated with CAT@HA-HMME NPs to investigate intracellular internalization. Before the incubation, CT26 exhibit unobvious fluorescent signals (Fig. 5b). After the incubation, both fluorescent signal regions (Fig. 5b) and the intensity (Fig. 5c) go up with the increased time from 0.5 to 6 h, suggesting that CAT@HA-HMME NPs can be successfully endocytosed into the cancer cells and then disassembled into fragments with higher fluorescence. To further investigate the effect of HA-CD44 interaction on the endocytosis of CAT@HA-HMME NPs, CAT@HA-HMME NPs were used to incubate several groups of CD44-positive CT26 (Fig. 5d) and SK-OV3 (Fig. S5c) for 6 h, where one group CT26 or SK-OV3 was pre-treated with HA. Then, these incubated CT26 and SK-OV3 were stained by DAPI and then investigated by confocal laser scan microscopy (CLSM). For HA pre-treated CT26 group, there is a few red fluorescence around the cell nucleus, indicating the few CAT@HA-HMME NPs in the cellular matrix. CT26 group without HA pre-treatment exhibits more red fluorescence signal from CAT@HA-HMME around cell nucleus (Fig. 5d). Meanwhile, the flow analysis was also used to quantitatively assess the uptake with or without HA treatment. The mean fluorescence intensity of CAT@HA-HMME NPs in the CT26 cells is 165-fold higher than that in the pre-treatment HA group (Fig. 5e). Similarly, SK-OV3 cells without HA pre-treatment exhibits stronger fluorescence signals than HA pre-treated group. This difference indicates that when CT26 and SK-OV3 are pretreated with HA, the cellular uptake of CAT@HA-HMME NPs can be partly blocked. Therefore, this fact implies that HA-CD44 interaction can be vital for the endocytosis of CAT@HA-HMME NPs.

Usually, the hypoxic tumor microenvironment (TME) [[43], [44], [45], [46]] would seriously weaken the effects of oxygen-dependent SDT. Herein, the encapsulated CAT catalyzes endogenous H_2_O_2_ to produce oxygen, probably relieving the hypoxic TME. To assess intracellular O_2_ level, two groups of CT26 cells were incubated with an oxygen indicator [Ru(dpp)_3_]^2+^Cl_2_ (whose red luminescence could be quenched by O_2_ [43,47]) under hypoxia conditions of 1% O_2_ for 24 h. Subsequently, only one group was then incubated with CAT@HA-HMME NPs solution. For the control group (CT26 cells without CAT@HA-HMME NPs treatment), there is strong red luminescence around the cell nucleus, indicating the presence of hypoxic TME (Fig. S5d, left panel). Interestingly, CT26 incubated with CAT@HA-HMME NPs show less red signal (Fig. S5d, right panel), suggesting the luminescent quenching of [Ru(dpp)_3_]^2+^Cl_2_ and indicating the efficient production of O_2_. Then, immunostaining was also used to investigate the HIF-1α expression in CT26 cells. The HA-HMME NPs incubated cells show strong green fluorescence signals (Fig. 5f), similar to the hypoxic conditions group (1% O_2_), indicating the expression of HIF-1α proteins. Conversely, after CAT@HA-HMME NPs treatments for 12 h, the cells exhibit negligible green fluorescence similar to that of the nomorxic conditions group (21% O_2_), suggesting the loaded alleviate hypoxia and reduce HIF-1α proteins expression. Thus, the alleviation of hypoxia is beneficial of the improvement of SDT.

**Fig. 6:**
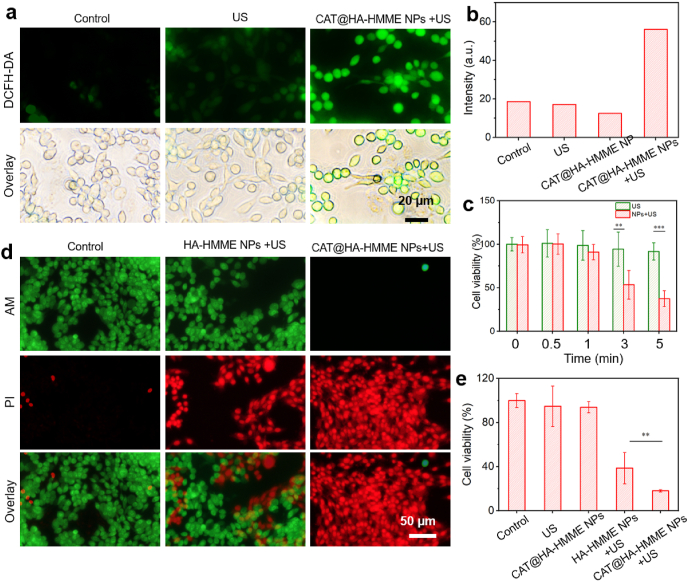
SDT *in vitro*. (a) Fluorescence images stained of SK-OV3 cells for intracellular ROS detection. (b) Semi-quantitative analysis of fluorescence signals of DCF based on the images shown in Fig. 6a. (c) Cell viability after different US exposure times. (d) Fluorescence images of SK-OV3 cells stained by PI and calcein-AM after different treatments. (e) Cell viability after different treatments.

Our previous reports have demonstrated that HMME can generate ^1^O_2_ *in vitro* under US irradiation [48], as shown in Figs. S6a and a strong green fluorescence indicates the ^1^O_2_ generation. To evaluate the *in vitro* ^1^O_2_ production ability by CAT@HA-HMME NPs, CT26 cells were incubated with 2′,7′-dichlorofluorescein diacetate (DCFH-DA) and divided into five groups (control, only US treatment, CAT@HA-HMME incubation, HA incubation + US treatment, CAT@HA-HMME incubation + US treatment). For three groups (control, US treatment, CAT@HA-HMME incubation, HA incubation + US treatment), there are very weak green fluorescence (Fig. S6b, Fig. 6a), indicating very low concentration ^1^O_2_ of in cells and/or inefficient production of ^1^O_2_ by only US treatment, CAT@HA-HMME incubation or HA + US incubation. On the contrary, CAT@HA-HMME + US group exhibits a strong green fluorescence, implying the mass production of intracellular ^1^O_2_ (Fig. 6a). The average fluorescence intensity of CAT@HA-HMME NPs + US is significantly the highest among these groups (Fig. 6b). These facts confirm that the disassembly of CAT@HA-HMME NPs in cells can generate ^1^O_2_ upon US, which should confer the toxic effect and achieve the therapeutic function afterward.

The sono-toxicity of CAT@HA-HMME NPs was studied using SK-OV3 cells as a CD44-positive model. Firstly, SK-OV3 cells incubated without and with CAT@HA-HMME were irradiated by US for different times (0–5 min), and the cell viabilities were analyzed by CCK-8 (Fig. 6c). The only US group maintains a high cell viability (>91.8%) during the entire 5 min, indicating negligible cytotoxicity from only US treatment. In contrast, the cell viability in CAT@HA-HMME + US group goes down from 99.5% at 0 min to 37.5% at 5 min, featuring greatly suppressed cell proliferation and massive cell death, which are also US irradiation duration-dependent. Then, to investigate the effect of CAT on the improvement of SDT, CAT@HA-HMME NPs or HA-HMME NPs solution were incubated with SK-OV3 cells for 6 h. Part of the groups were treated with US (2.5 W/cm^2^) for 3 min. The cell apoptosis in five groups (control (I), US treatment (II), CAT@HA-HMME NPs incubation (III), HA-HMME NPs incubation + US treatment (IV), CAT@HA-HMME NPs incubation + US treatment (V)) was analyzed by calcein-AM (live cells)/propidium iodide (PI, dead cells) co-staining (Figs. 6d and S7) and CCK-8 assay (Fig. 6e). It can be visualized that group I-IV exhibit strong green fluorescence (live cells) and negligible red fluorescence (died cells). Group IV shows less green fluorescence and more red fluorescence, indicating more dead cells. Importantly, group V reveals almost all dead cells. Furthermore, the therapeutic effect of SDT was quantitatively assessed by using CCK-8 (Fig. 6e). Both group II and group III retain high cell viability (i.e. 94.8% and 93.9%, respectively). However, the cell viabilities of group IV-V sharply decrease to 38.6% and 18.1% after US irradiation. Meanwhile, there is a significant level (**P < 0.01) between group IV and group V. These results demonstrate that ROS produced by HA-HMME NPs under US could induce cancer cell ablation, and the encapsulated CAT can assist O_2_-dependant SDT due to the decomposition of endogenous H_2_O_2_ into O_2_.

**Fig. 7:**
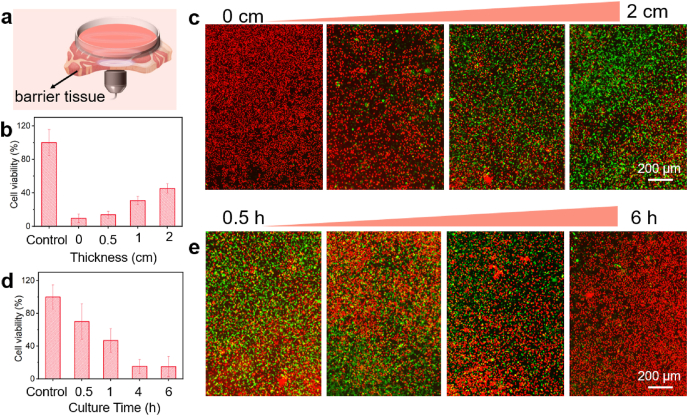
Effects of different barrier-thickness and culture-time on SDT. (a) Schematic illustration of deep-tissue SDT employing lean pork as the barrier. Cell viability (b) and Calcein AM/PI staining fluorescence images (c) of cells with different thicknesses (0–2 cm) of the barrier. Cell viability (d) and Calcein AM/PI staining fluorescence images (e) of cells at different culture times.

To investigate deep-tissue SDT, the pork is used to imitate tissue barriers (Fig. 7a). The effect of tissue thicknesses on cell viability was studied by CCK-8 and Calcein-AM/PI co-staining. CAT@HA-HMME NPs solution was used to incubate SK-OV3 cells for 6 h. The treated cells were covered with pork and then irradiated by US for 3 min in the absence or presence of pork barrier with different thicknesses. Clearly, with the increased pork thickness from 0 to 2 cm, the cell viabilities increase from ∼9.5% to 45.3% (Fig. 7b). To visualize SDT therapy vividly, the treated cells were co-stained with calcein-AM and PI. As shown in Fig. 7c, the cells almost exhibit the strong red fluorescence without a barrier, indicating that all are dead cells. When the thickness of the barrier changes from 0.5 to 2 cm, the green area gradually becomes larger while the red area becomes less, revealing the increased number of live cells and the decreased dead cells. Thus, one can deduce that US can penetrate the pork barrier (2 cm) to trigger SDT effect of CAT@HA-HMME NPs, and SDT effect may be further improved by prolonging the irradiated time or heightening the power.

To study the effects of culture time (namely disassembly time), SK-OV3 cells were cultured with CAT@HA-HMME NPs for different times (0.5–6 h), and then executed US irradiation for 3 min. According to the CCK-8 assay, the cell viabilities decrease from 70.0% to 15.0% when the culture time increases from 0.5 to 6 h (Fig. 7d). Meanwhile, the cells were stained with calcein-AM/PI to visualize SDT efficacy (Fig. 7e). When the culture time was 0.5 h, the cells with red fluorescence are relatively less. After prolonged culture time, the cells with red signals become significantly increase. And when the time reaches 6 h, the red fluorescence overwhelmingly occupies the whole image. Therefore, a good therapeutic effect can be achieved by prolonging the culture time, which would be attributed to the simultaneous accumulation/endocytosis and HAase-induced disassembly of CAT@HA-HMME NPs in the intracellular.

### 3.4 Blood-circulation and SDT *in vivo*

**Fig. 8:**
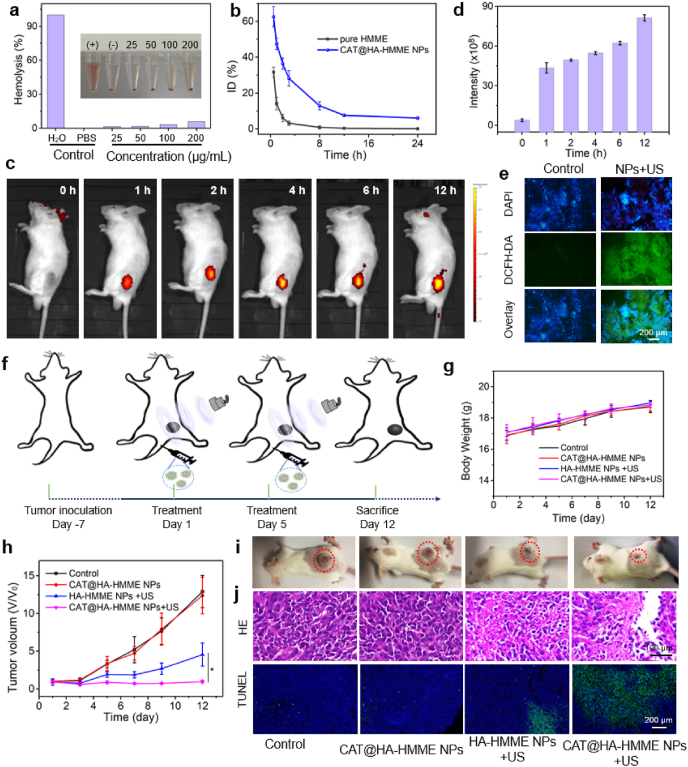
SDT *in vivo*. (a) The hemolysis rates of CAT@HA-HMME NPs with different concentrations (0–200 μg/mL). (b) Blood clearance rate of CAT@HA-HMME NPs and pure HMME in mice over 24 h after intravenous injection. (c) Fluorescence images of a representative mouse. (d) The fluorescence intensity of tumor site. (e) Ex-vivo ROS fluorescence images of DCFH-DA stained tumor slices after different treatments. (f) Schematic illustration of the treatment plan. Body weight (g) and tumor volume (h) of mice during 12 days. (i) Photos of mice. (j) H&E and TUNEL staining of tumor slides.

For further animal experiments, the hemolysis assay was firstly measured by using animal blood [49]. The blood of mice was taken out and equally divided into six parts. One was mixed with water as a positive control group (+), one was mixed with PBS as a negative control group (−); and others were mixed with CAT@HA-HMME NP solution (25–200 μg/mL). After incubation for 1 h, the absorbances at 576 nm of all the groups were investigated by UV–vis spectrometer. The color of supernatant in the positive group is relatively red, implying very severe hemolysis (Fig. 8a). The hemolysis ratio of the positive group is set as 100% and that of the negative group is set as 0%. The color of supernatant in sample groups gradually turns slightly reddish but remains transparent. And the hemolysis ratio is <5.8% even at 200 μg/mL, indicating the good hemocompatibility of CAT@HA-HMME NPs. The components of CAT@ HA-HMME NPs are FDA-approved HMME, clinical HA, and biologically derived CAT, which offer NPs good biocompatibility. Therefore, CAT@HA-HMME NPs could be administered for *in-vivo* treatment.

To study long retention ability, mice were intravenously injected with CAT@HA-HMME NPs or pure HMME, and their blood was taken out at different time points for pharmacokinetic assay (Fig. 8b). The concentrations of HMME in the blood were measured by fluorescence spectroscopy [26]. For the pure HMME, the concentration of HMME decreases rapidly to 31.68% ID at 0.5 h post i.v. injection, indicating quick clearance and a short half-life (t_1/2_ = 0.82 h). Importantly, when CAT@HA-HMME NPs solution is injected into mice, the concentration of HMME goes down slowly and it can maintain 6.09% ID even at 24 h, showing a long blood-circulation half-life time (t_1/2_ = 4.17 h). This long half-life time should be attributed to the suitable size for longer blood circulation, which makes CAT@HA-HMME NPs have the potential to accumulate at the tumor site.

The prerequisite for SDT is the high accumulation of sonosensitizers at the tumor site and the effective generation of ROS under US irradiation. To evaluate the targeting accumulation of CAT@HA-HMME NPs, CAT@HA-HMME NPs were pre-labeled by Cy5.5 and tracked by a non-invasive optical imaging technique. After the administration of CAT@HA-HMME NPs, the tumor region is gradually brightened (Fig. 8c), and the relative signal intensity increase from 3.93 at 0 h to 81.3 at 12 h (Fig. 8d). It is well established that CAT@HA-HMME NPs can effectively accumulate at the tumor site, due to HA-targeting ability. Next, to reveal ROS generation *in vivo*, two groups of mice are injected with CAT@HA-HMME NPs, where only one group was irradiated by US for 10 min. The tumors were collected after treatments for DCFH-DA staining (Fig. 8e). As expected, when only treated with CAT@HA-HMME NPs, no significant ROS fluorescence is found. Interestingly, the CAT@HA-HMME NPs + US group shows significant signals, implying the US-induced ROS generation ability in the tumoral region. Therefore, CAT@HA-HMME NPs are active for targeting the cancer cells and generate ROS upon the US for SDT.

Motivated by the ability of ROS generation *in-vitro/in-vivo* and accumulation in tumor, the SDT efficacy of CAT@HA-HMME NPs *in vivo* was monitored on CT26 tumor model for 12 days (Fig. 8f). The tumor-bearing Balb/c mice were randomly divided into four groups: (1) Control (PBS, 100 μL), (2) CAT@HA-HMME NPs (100 μL, 9.8 mg/kg), (3) HA-HMME NPs (100 μL,9.8 mg/kg) + US (1.0 MHz, 1.75 W/cm^2^, 10 min, 25% duty cycle), (4) CAT@HA-HMME NPs (100 μL, 9.8 mg/kg) + US. The treatment was repeated twice on day 1 and day 5. During the whole treatment, all mice show negligible weight changes (Fig. 8g), suggesting the HA-HMME NPs/CAT@HA-HMME NPs and US are reasonably safe. The HA-HMME NPs + US group shows a certain antitumor effect, and CAT@HA-HMME NPs + US has a better inhibitory effect (*p < 0.05, Fig. 8h). This comparison may be attributed to the fact that CAT encapsulated in the nanoparticles can relieve the hypoxia TME and produce a higher amount of ROS. In addition, the therapeutic efficacy was further validated by the images of hematoxylin and eosin (H&E) [19] and terminal deoxynucleotidyl transferase deoxyuridine triphosphate (dUTP) nick end labeling (TUNEL) stained (Fig. 8i). As excepted, HA-HMME NPs + US and CAT@HA-HMME NPs + US caused more severe damages to tumor cells, and their levels of cell apoptosis were significantly higher than that of control groups (group 1–2), which was consistent with the trends in the tumor inhibition. Meanwhile, there are no obvious toxic side effects in major organs (Fig. 8j) due to the good biosafety of CAT@HA-HMME NPs. Such results confirmed a desirable therapeutic effect of HA-HMME NPs-mediated SDT and enhancement of SDT through CAT encapsulation.

## 4 Conclusions

In summary, we have reported an on-demand assembly strategy for longer-blood-circulation and disassembly in tumors for boosting SDT. Polymeric nanoparticles (CAT@HA-HMME NPs) as a model have been prepared by the first conjugation of HMME and HA and then the subsequent CAT-encapsulation/assembly process. After an aqueous solution of CAT@HA-HMME NPs is intravenously injected in mice, five favorable features can be obtained as follow. (I) The CAT@HA-HMME NPs have suitable sizes (∼80 nm), which confers longer blood half-time (t_1/2_ = 4.17 h, higher than 0.82 h of HMME molecule). Simultaneously, the encapsulation in nanoparticles cannot only prevent CAT leakage but also provide a shield for CAT during blood circulation, protecting it from the damage of body proteases. (II) The presence of HA in CAT@HA-HMME NPs is specifically accumulated within cancer cells via CD44-HA recognition, resulting in high retentions in the tumor site. (III) Endogenous hyaluronidases (HAase) induce the *in-situ* disassembly of CAT@HA-HMME NPs in tumor, leading to the release of HA-HMME and CAT. (IV) The released HA-HMME exhibit stronger photoabsorption and fluorescence as well as higher ^1^O_2_ generation ability than CAT@HA-HMME NPs, due to disassembly-induced disruption of ACQ effect. Meanwhile, the released CAT depletes endogenous H_2_O_2_ into O_2_ for relieving the hypoxic TME, efficiently boosting the production of cytotoxic ^1^O_2_ for SDT. (V) As a result, CAT@HA-HMME NPs can activate more ^1^O_2_-triggered irreversible oxidation of cancer cells through double enhancement, resulting in the internal physiological and metabolic dysfunction and inhibition of tumor growth. Therefore, the present CAT@HA-HMME NPs have great potential for future practical applications in SDT of tumor. More importantly, this work provides some insight into the design and development of other nanoagents with the assembly for longer-blood-circulation and disassembly in tumor for boosting therapy effects.

## Declaration of competing interest

The authors declare that they have no known competing financial interests or personal relationships that could have appeared to influence the work reported in this paper.

## CRediT authorship contribution statement

**Mei Wen:** Investigation, Data curation, Writing – original draft. **Nuo Yu:** Investigation, Writing – original draft. **Shiwen Wu:** Investigation. **Mengmeng Huang:** Formal analysis. **Pu Qiu:** Formal analysis. **Qian Ren:** Investigation. **Meifang Zhu:** Writing – review & editing. **Zhigang Chen:** Funding acquisition, Writing – review & editing.
