# Supplementary material for: On-demand assembly of polymeric nanoparticles for longer-blood-circulation and disassembly in tumor for boosting sonodynamic therapy
Source: Bioact Mater. 2022 Mar 12;18:242–53. doi: 10.1016/j.bioactmat.2022.03.009 (PMC8961299; doi:10.1016/j.bioactmat.2022.03.009)
Supplement: Multimedia component 1 [file mmc1.docx]

Supporting Information

On-demand assembly of polymeric nanoparticles for longer-blood-circulation and disassembly in tumor for boosting sonodynamic therapy

Mei Wen, Nuo Yu, Shiwen Wu, Mengmeng Huang, Pu Qiu, Qian Ren, Meifang Zhu, Zhigang Chen*

State Key Laboratory for Modification of Chemical Fibers and Polymer Materials, College of Materials Science and Engineering, Donghua University, Shanghai 201620, China

**Corresponding Author**

*E-mail: [zgchen@dhu.edu.cn](mailto:zgchen@dhu.edu.cn) (Z.G. Chen)

**Experiments**

**Cell viability.** To study the biosafety, CAT@HA-HMME NPs (0-200 μg/mL) were incubated with SK-OV3, CT26, and HUVEC cells in darkness for for 24 h and the cell viabilities were tested using the standard CCK-8 assay. For comparison, the HA (0-200 μg/mL) and HMME (0-50 μg/mL) were also incubated with SK-OV3 under the same conditions for 24 h. All experiments were performed four times, independently.

**Figures**


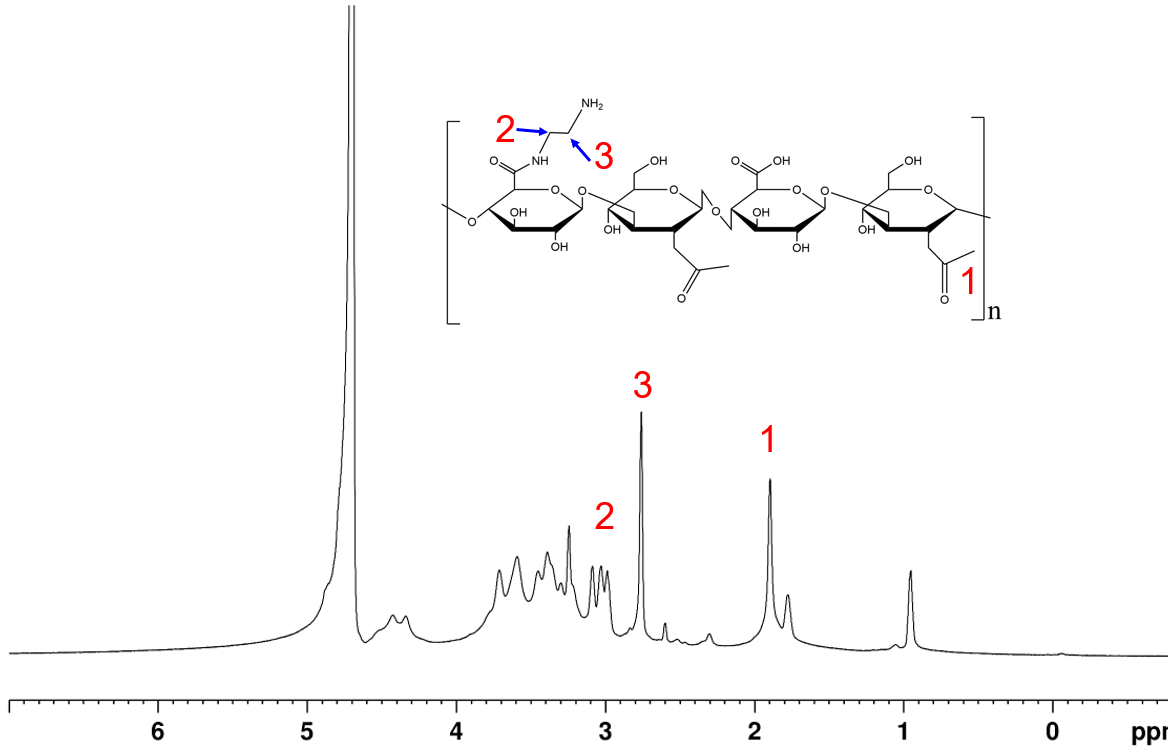


**Figure S1.** ^1^H NMR spectrum of HA-NH_2_.


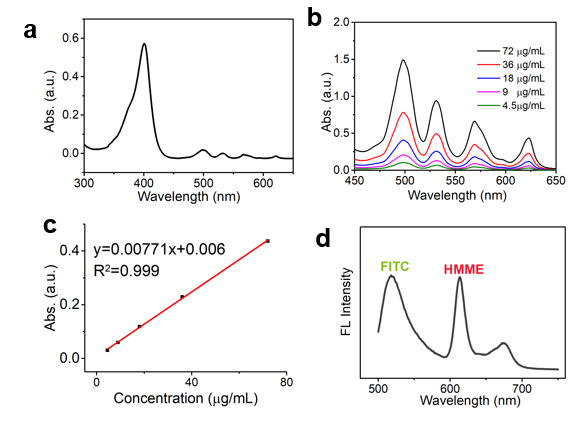


**Figure S2.** (a) Photographs of CAT@HA-HMME NPs in different biological fluids. (b) UV-vis spectrum of HMME of different concentrations of HMME. (c) Standard cure of HMME. (d) Fluorescence spectrum of FITC labeled CAT@HA-HMME NPs.


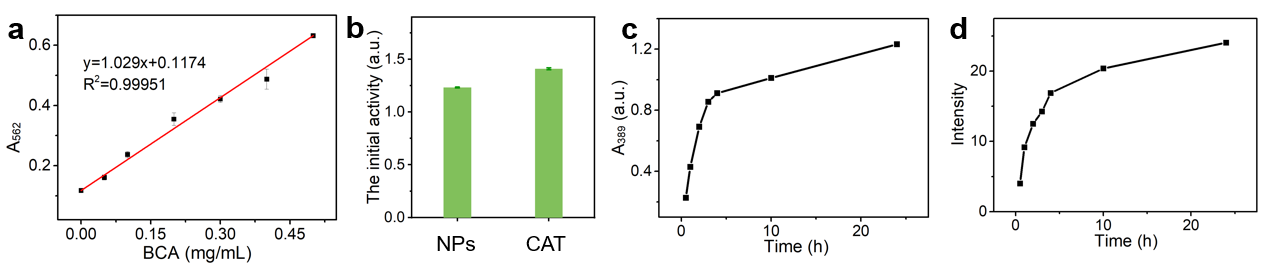


**Figure S3.** (a) Standard curve of BCA. (b) The intal catalase activity. Absorption change (c) and (d) Fluorescence intensity change over a period of 24 h.


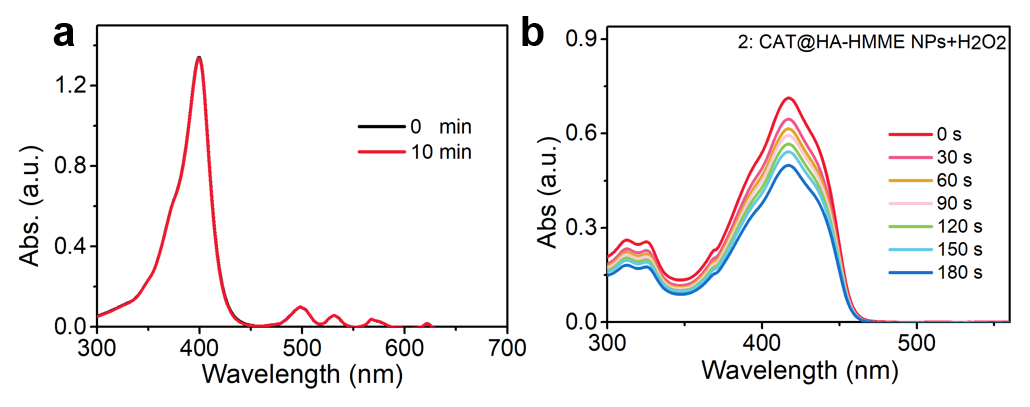


**Figure S4.** (a) UV-vis spectra of CAT@HA-HMME upon US irradiation. (b) Time-dependent oxidation of DPBF in CAT@HA-HMME+HAase solution.


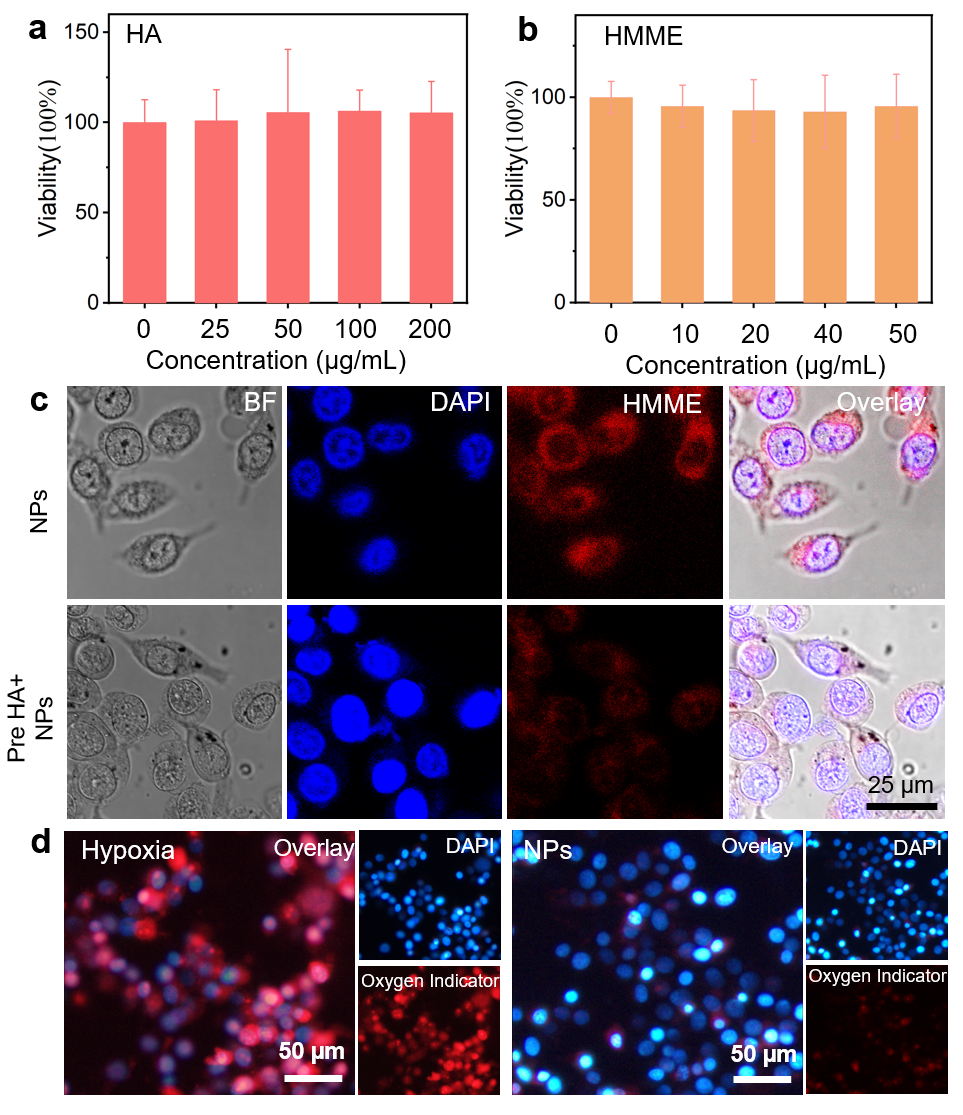


**Figure S5.** Cell viability of SKOV3 cells with free HA (a) and HMME (b) at different concentrations for 24 h. (c) Confocal images of SK-OV3 cells incubated with CAT@HA-HMME. (d) Luminescence images of CT26 without or with CAT@HA-HMME NPs incubation for showing hypoxia. Cell nuclei (blue), intracellular O_2_ level (red).


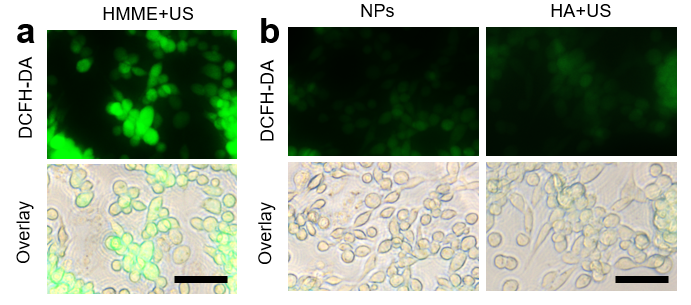


**Figure S6.** ROS detection of SK-OV3 cells treated with HMME+US (a), NPs and HA+US (b). Scar bar is 50 µm.


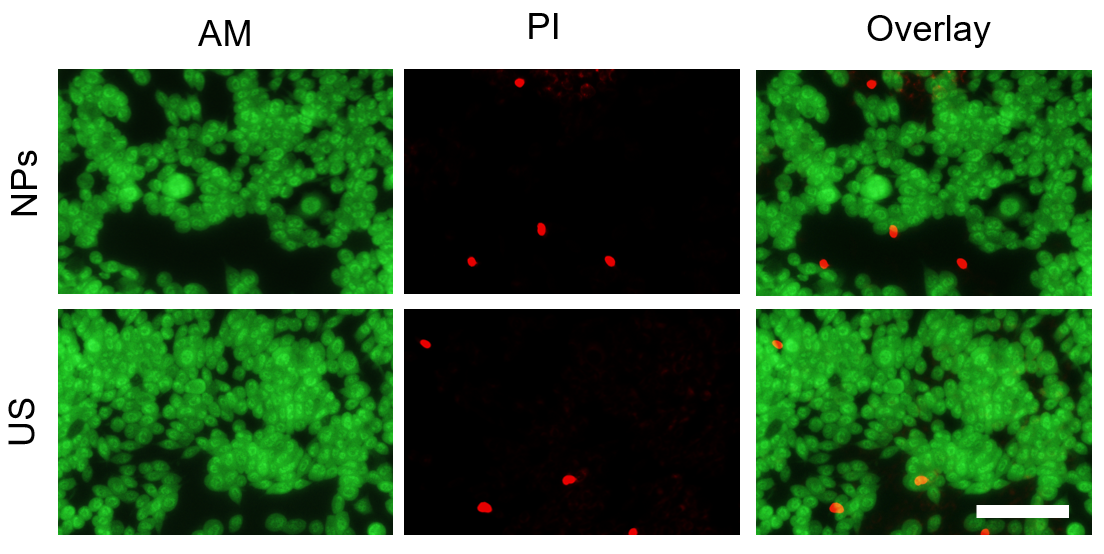


**Figure S7.** Fluorescence images of SK-OV3 cells stained by PI and calcein AM after different treatments. Scar bar is 50 µm.


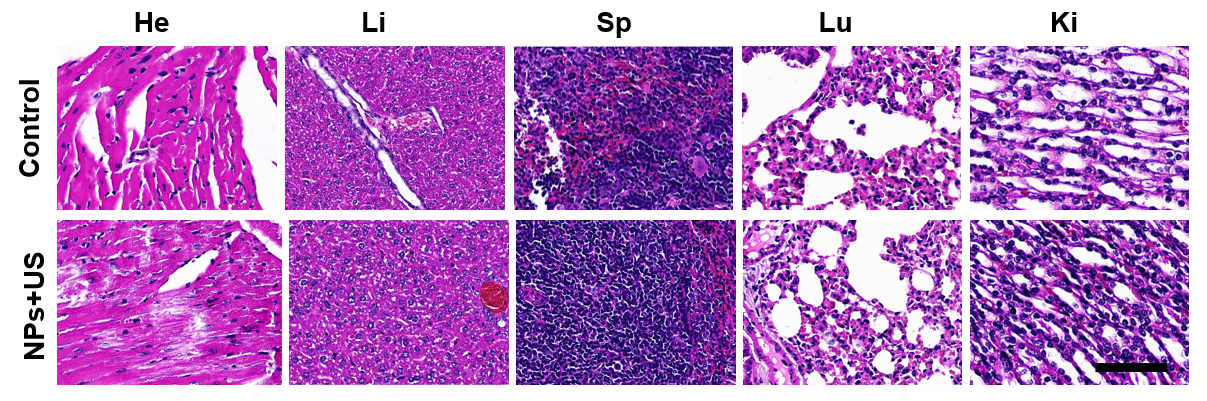


**Figure S8.** Histological photos of major organs. Scar bar is 100 µm.
